# Identification of heterogenous nuclear ribonucleoproteins (hnRNPs) and serine- and arginine-rich (SR) proteins that induce human papillomavirus type 16 late gene expression and alter L1 mRNA splicing

**DOI:** 10.1007/s00705-021-05317-2

**Published:** 2021-12-03

**Authors:** Chengyu Hao, Lijing Gong, Xiaoxu Cui, Johanna Jönsson, Yunji Zheng, Chengjun Wu, Naoko Kajitani, Stefan Schwartz

**Affiliations:** 1 Department of Laboratory Medicine, Lund University, BMC-B13, 221 84 Lund, Sweden; 2 China Institute of Sport and Health Sciences, Beijing Sport University, Haidian District, Beijing, People’s Republic of China; 3 School of Biomedical Engineering, Dalian University of Technology, Dalian, 116024 Liaoning Province People’s Republic of China; 4 Department of Medical Biochemistry and Microbiology, Uppsala University, BMC-B9, 751 23 Uppsala, Sweden

## Abstract

We have determined the effect of seven serine- and arginine-rich (SR) proteins and 15 heterogenous nuclear ribonucleoproteins (hnRNPs) on human papillomavirus type 16 (HPV16) late gene expression. Of the seven SR proteins analyzed here, SRSF1, SRSF3, and SRSF9 induced HPV16 late gene expression, and five of the SR proteins affected HPV16 L1 mRNA splicing. Of the 15 hnRNP proteins analyzed here, hnRNP A2, hnRNP F, and hnRNP H efficiently induced HPV16 late gene expression, and all of the hnRNPs affected HPV16 L1 mRNA levels or mRNA splicing. Thus, the majority of SR proteins and hnRNPs have the potential to regulate HPV16 L1 mRNA splicing. Strict control of the expression of the immunogenic L1 and L2 capsid proteins may contribute to the ability of HPV16 to cause persistence.

**Supplementary Information:**

The online version contains supplementary material available at 10.1007/s00705-021-05317-2.

Persistent infections with a subset of human papillomaviruses (HPVs) defined as high-risk HPVs may cause cervical cancer [2, 21]. HPV16 dominates among the mucosal HPV types and is present in approximately 50% of all cervical cancers [24]. The ability of HPV16 to control expression of the highly immunogenic late HPV16 genes L1 and L2 may contribute to immune evasion by HPV16 [3, 5, 8, 14]. The HPV16 late L1 and L2 mRNAs utilize multiple HPV16 splice sites during mRNA processing, most importantly SD880, SA3358, SD3632, and SA5639 (Supplementary Fig. 1A and C), of which SD3632 and SA5639 are unique to the late L1 mRNAs. HPV16 has the ability to establish persistent infections in its host that may progress to cancer. Strict control of expression of the highly immunogenic L1 and L2 capsid proteins may contribute to this property. It is therefore of interest to identify factors that control HPV late gene expression. HPV16 splice sites are controlled by cellular factors, many belonging to the serine-arginine-rich (SR) protein family or to the heterogeneous nuclear ribonuclear protein (hnRNP) family [9, 10]. Here, we have compared the effect of various SR proteins and hnRNP proteins on HPV16 late gene expression and L1 alternative splicing in the same experimental system.

HeLa cells were transfected with plasmids encoding seven serine- and arginine-rich (SR) proteins together with an HPV16-derived subgenomic reporter plasmid named pC97ELsL (Supplementary Fig. S1B). The use of HeLa cells in this study is justified by the fact that they are HPV18 positive and as such represent cells that are targeted by HPV. Methods are detailed in the Supplementary Information file. HeLa cells were cultured in Dulbecco’s modified Eagle medium (GE Healthcare Life Science Hyclone Laboratories) with 10% bovine calf serum and 100 U of penicillin and 100 µg of streptomycin (Gibco Thermo Fisher Science) per ml. The plasmid pC97ELsL has a secreted luciferase (sLuc) reporter gene inserted after a poliovirus 2A internal ribosome entry site (IRES) in the L1 coding region that serves as a marker for HPV16 late gene expression (Supplementary Fig. S1B and C) [12, 13]. Transfections were carried out using Turbofect according to the manufacturer’s instructions (Thermo Fisher Science). Each plasmid was used for transfection in a minimum of three independent experiments. sLuc activity in the cell culture medium at 20 h posttransfection was determined using a “Ready To Glow” secreted luciferase reporter assay according to the instructions of the manufacturer (Clontech Laboratories). The results revealed that SRSF1, SRFS3, and SRSF9 induced HPV16 late gene expression (Fig. 1A). This is consistent with previously published results [11, 22, 23]. Transfections with pC97ELsL together with a serially diluted SR protein expression plasmid revealed that induction of HPV16 late gene expression by SRSF1 was saturated at relatively low levels of transfected SRSF1 plasmid (Fig. 1B). Induction of HPV16 late gene expression by SRSF3 and SRSF9 was concentration-dependent, but in a different manner for the two proteins (Fig. 1B). While induction of HPV16 late gene expression by SRSF3 was optimal at 250 ng of transfected plasmid, induction of HPV16 late gene expression by SRSF9 was optimal at the highest levels of transfected plasmid (2 µg) (Fig. 1B). These results suggested that SRFS3 and SRSF9 may differ in their control of HPV16 late mRNA splicing. SR proteins are modular, consisting of an RNA-binding region and an RS-domain that is responsible for protein-protein interactions. SRp30DRS produces an SRSF9 protein lacking the RS domain. Since SRp30DRS activates sLuc production, albeit to a lower extent than SRSF9, the RS domain is not absolutely required for induction of HPV16 late gene expression (Fig. 1B). To determine how the SR proteins affect HPV16 late mRNA splicing, we first extracted total RNA from transfected cells using TRI Reagent and a Direct-zol RNA MiniPrep Kit (ZYMO Research) according to the manufacturer’s protocol. One μg of total RNA was reverse transcribed in a 20-μl reaction mixture at 37 °C using M-MLV Reverse Transcriptase and random primers (Invitrogen) according to the protocol of the manufacturer. One microliter of cDNA was subjected to PCR amplification with the HPV16-L1-mRNA-specific RT-PCR primers 773S and L1A (for primer location on the HPV16 mRNAs, see Supplementary Fig. S1C, and for primer sequences, see Supplementary Table S1). The results revealed that SRSF3 and SRSF9, and to a lesser degree SRSF1, enhanced production of late L1 mRNAs relative to pC97ELsL transfected with empty pUC plasmid (-) (Fig. 1C). Interestingly, SRSF3 overexpression strongly shifted HPV16 L1 alternative splicing to L1 mRNAs containing the central exon located between SA3358 and SD3632 (Fig. 1C), whereas SRSF9 induced skipping of this exon to favor an L1 mRNA that is spliced between SD880 and SA5639 (Fig. 1C). Since both SRSF3 and SRFS9 induced HPV16 late gene expression, there is no correlation between HPV16 late gene expression and the absence or presence on the L1 mRNAs of the small central exon between SA3358 and SD3632. Since SRSF9 caused skipping of this central exon, SRSF9 may have a splicing inhibitory function on inclusion of the exon between SA3358 and SD3632.

**Fig. 1:**
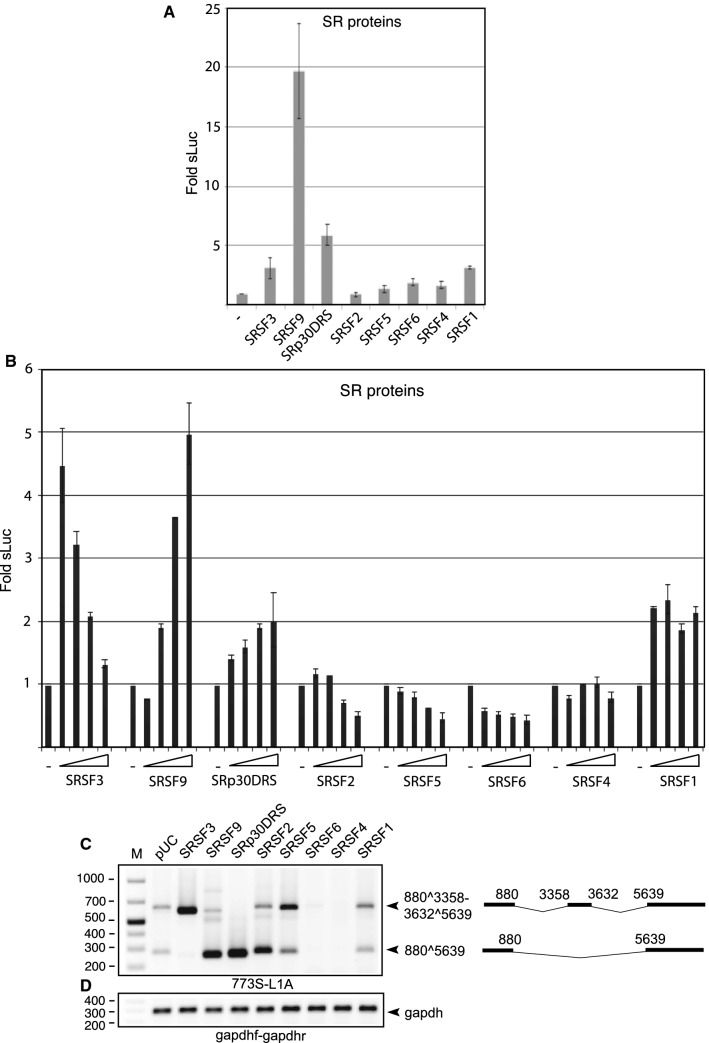
(A) Secreted luciferase enzyme activity (sLuc) in the cell culture medium at 24 h after transfection of HeLa cells with pC97ELsL and plasmids expressing the indicated SR proteins or serial dilutions of the indicated plasmids (2.0, 1.0, 0.5, and 0.25 µg). SRp30DRS is a deletion mutant of SRSF9 in which the RS domain has been deleted [23]. (B) Mean values and standard deviations of triplicate transfections are shown. (C) RT-PCR with primers 773S and L1A on total RNA extracted from HeLa cells transfected with the indicated plasmids. Schematic representations of the alternatively spliced HPV16 L1 mRNAs detected by RT-PCR are shown to the right of the gel image. (D) gapdh cDNA was amplified by PCR using the primers gapdhf and gapdhr. M, molecular weight marker; pUC, pUC vector

The deletion also did not affect the ability of SRSF9 to promote skipping of the central exon between SA3358 and SD3632 on the L1 mRNAs (Fig. 1C). We therefore conclude that SRSF3 and SRSF9 enhanced HPV16 late gene expression but affected HPV16 L1 mRNA splicing differently. SRp30DRS retained the ability to promote exon skipping, indicating that this property of SRSF9 is independent of the RS domain. The two other SR proteins that affected alternative splicing of the L1 mRNAs, SRSF2 and SRSF5, either promoted exclusion (SRSF2) or inclusion (SRSF5) of the central exon between SA3358 and SD3632 compared to cotransfection with empty pUC plasmid (Fig. 1C). Despite the effect of SRSF2 and SRSF5 on alternative splicing of HPV16 L1, neither of these two proteins induced HPV16 late gene expression (Fig. 1A and B). Finally, we were surprised to see that overexpression of SRSF4 and SRSF6 strongly reduced the levels of HPV16 late L1 mRNAs (Fig. 1C).

We also performed RT-PCR on RNA extracted from HeLa cells transfected with pC97ELsL and serial dilutions of expression plasmids encoding SR proteins. Of the three proteins that induced HPV16 late gene expression, monitored by measuring sLuc as shown in Figure 2A and B (SRSF1, SRSF3, and SRSF9), SRSF1 promoted inclusion of the SA3358-SD3632 exon on the L1 mRNAs (Fig. 2A), and so did SRSF3 (Fig. 2B), even at low levels of transfected SRSF3 plasmid, while overexpression of SRSF9 caused exclusion of the same exon in a plasmid-concentration-dependent manner (Fig. 2C). Of the three SR proteins – SRSF2, SRSF4 and SRSF5 – that did not induce HPV16 late gene expression (monitored by measuring sLuc as shown in Figure 1A and B), SRSF2 promoted inclusion of the SA3358-SD3632 exon on the L1 mRNAs (Fig. 2D), while overexpression of SRSF4 resulted in a dose-dependent reduction of both alternatively spliced L1 mRNAs (Fig. 2E). SRSF5 promoted inclusion of the SA3358-SD3632 exon on the L1 mRNAs in a manner similar to SRSF1, but less efficiently than SRSF3 (Fig. 2F). In conclusion, five SR proteins either promoted exon inclusion or skipping on the HPV16 late L1 mRNAs, but only three SR proteins induced HPV16 late gene expression at the protein level.

**Fig. 2:**
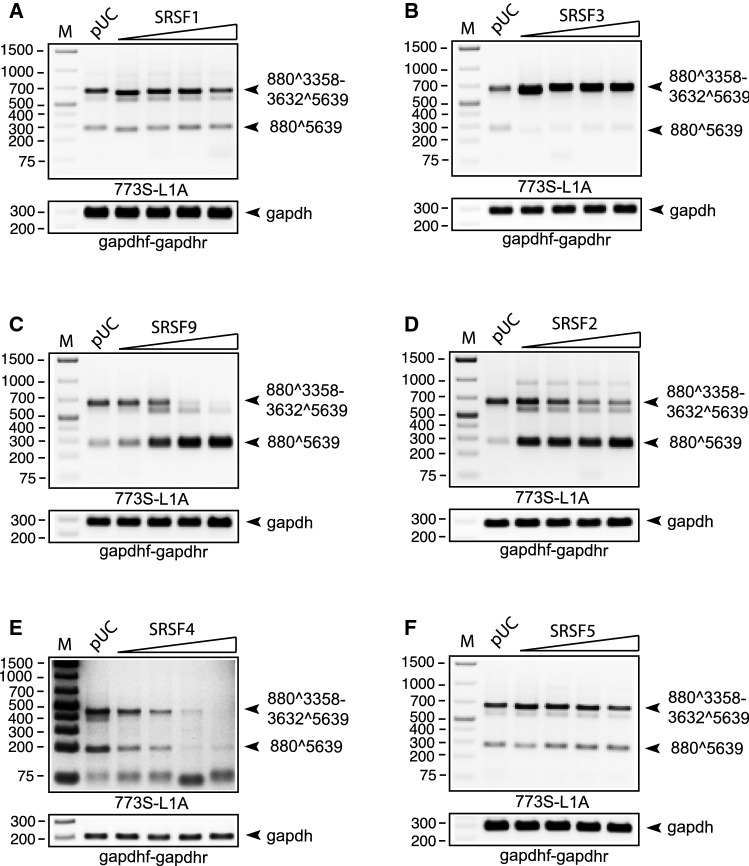
(A-F) RT-PCR with primers 773S and L1A on RNA extracted from HeLa cells transfected with pC97ELsL in the presence of empty pUC plasmid or 2-fold serially diluted SR-protein expression plasmid (2.0, 1.0, 0.5, and 0.25 µg). Splicing of the HPV16 mRNA represented by the PCR-amplified cDNAs is indicated to the right of each gel image. M, molecular weight marker

Next, we compared the effect of various hnRNPs on HPV16 late gene expression and HPV16 late L1 mRNA splicing. pC97ELsL was individually co-transfected with each of fifteen hnRNP expression plasmids, and sLuc levels were determined (Fig. 3A and B). As can be seen in Figure 3A and B, at least three hnRNPs induced HPV16 late gene expression: hnRNP A2, hnRNP F, and hnRNP H. RT-PCR with primers 773S and L1A revealed that hnRNP proteins affected the HPV16 L1 mRNAs in different ways (Fig. 3C). hnRNP A1, hnRNP AB, hnRNP G, hnRNP K, and hnRNP L caused exclusion of the same exon from the L1 mRNAs (Fig. 3C), whereas hnRNP A2, hnRNP C, hnRNP E1, hnRNP F, hnRNP H, hnRNP Q, and hnRNP R promoted inclusion of the exon between SA3358 and SD3632 on the L1 mRNAs (Fig. 3C). We have previously reported that hnRNP C and hnRNP G affect HPV16 L1 mRNA splicing in a manner that was confirmed here [4, 25]. Overexpression of hnRNP D and hnRNP DL appeared to have an inhibitory effect on the L1 mRNAs, which is in line with previously reported results on hnRNP D and L1 mRNA splicing (Fig. 3C) [13]. We conclude that the majority of the hnRNPs analyzed here affected HPV16 L1 mRNA levels or HPV16 L1 mRNA splicing, while only three hnRNPs significantly induced HPV16 late gene expression.

**Fig. 3:**
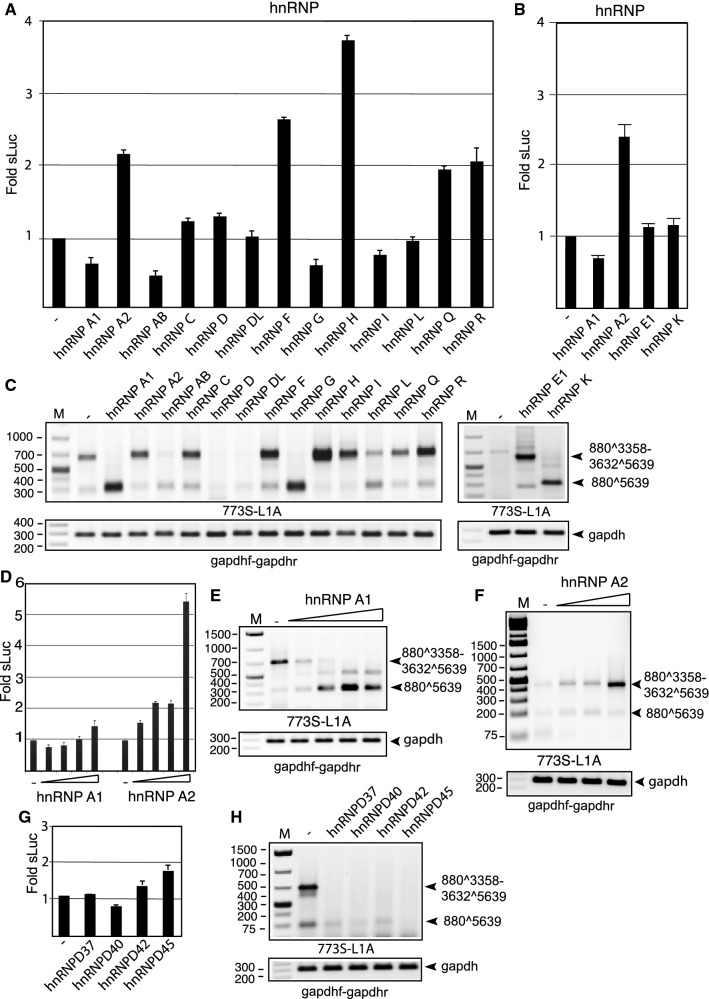
(A, B, D, and G) Secreted luciferase enzyme activity (sLuc) in the cell culture medium at 24 h after transfection of HeLa cells with pC97ELsL and expression plasmids encoding the indicated hnRNP proteins. Mean values and standard deviations of triplicate transfections are shown. (D) hnRNP A1 and hnRNP A2 plasmids were serially diluted 2-fold prior to transfection (2.0, 1.0, 0.5, and 0.25 µg). (C, E, F, H) RT-PCR with primers 773S and L1A on total RNA extracted from HeLa cells transfected with the indicated plasmids. (E) The hnRNP A1 plasmid was serially diluted 2-fold prior to transfection (2.0, 1.0, 0.5, and 0.25 µg). (F) The hnRNP A2 plasmid was serially diluted 2-fold prior to transfection (1.0, 0.5, and 0.25 µg). gapdh cDNA was amplified by PCR using the primers gapdhf and gapdhr. M, molecular weight marker; -, transfection with empty CMV-promoter-containing plasmid

hnRNP A1 and A2 have previously been shown to affect splicing of the E6 and E7 mRNAs in an opposite manner [27]. We therefore transfected cells with a serial dilution of hnRNP A1 or A2 together with pC97ELsL to confirm the effects of hnRNP A1 and A2 on alternative splicing of the HPV16 L1 mRNAs. sLuc levels confirmed that hnRNP A2 primarily induced late gene expression and that hnRNP A1 only had a minor effect on HPV16 late gene expression levels (Fig. 3D). RT-PCR revealed that hnRNP A1 altered L1 mRNA splicing and promoted exclusion of the SA3358-SD3632 central exon in a dose-dependent manner (Fig. 3E), whereas hnRNP A2 promoted inclusion of the same exon (Fig. 3F). Thus, in addition to the opposite effects of hnRNP A1 and A2 on HPV16 E6 and E7 mRNA splicing, hnRNP A1 and hnRNP A2 had opposite effects on alternative splicing of the HPV16 late L1 mRNAs.

The hnRNP D gene gives rise to four alternatively spliced mRNAs encoding hnRNP D proteins with molecular weights of 37, 40, 42, and 45 kDa (hnRNP D37, D40, D42, and D45) [20]. These alternatively spliced hnRNP D mRNAs are differentially expressed in various tissues and show differences in activity [20]. For example, HeLa cells produce more hnRNP D40 and hnRNP D45 mRNAs than hnRNP D37 and hnRNP D42 mRNAs [13]. We transfected cells with hnRNP D plasmids expressing hnRNP D37, D40, D42, or D45 together with HPV16 reporter plasmid pC97ELsL and monitored sLuc levels. None of the hnRNP D proteins significantly affected sLuc levels (Fig. 3G), but all hnRNP D proteins reduced HPV16 L1 mRNA levels (Fig. 3H). The discrepancy between sLuc levels and L1 mRNA levels could possibly be explained by splicing inhibition by the hnRNP D proteins and promotion of L1 mRNA production, as it would produce sLuc as well (Supplementary Fig. S1C). RT-PCR with L2-mRNA-specific primer levels revealed that L2 mRNAs were increased by overexpression of hnRNP D (Supplementary Fig. S1D), suggesting that hnRNP D proteins inhibited splicing and promoted production of unspliced L2 mRNAs that also produce sLuc, thereby compensating for the reduced L1 mRNA levels.

Finally, transfection of serially diluted plasmids encoding hnRNP Q or hnRNP R with pC97ELsL confirmed that overexpression of either of the proteins induced HPV16 late gene expression, albeit at relatively high levels of expression plasmid (Supplementary Fig. S2A and B). RNA analysis by RT-PCR confirmed that hnRNP Q and hnRNP R acted by enhancing inclusion of the central exon between SA3358 and SD3632 on the HPV16 L1 mRNAs (Supplementary Fig. S2C and D). Quantitation of the levels of the alternatively spliced L1 mRNA revealed that the L1 mRNAs with the SA3358-SD3632 exon retained (L1) increased to a higher degree than L1 mRNAs with the SA3358-SD3632 exon excluded (L1i) in response to hnRNP Q or hnRNP R (Supplementary Fig. S2E and F). L1i represents the L1 mRNA spliced directly from SD880 to SA5639. We also investigated if hnRNP Q or hnRNP R affected alternative splicing of early HPV16 mRNAs, including mRNAs utilizing the HPV16 splice sites SD226 and SA409, SA529, or SA742 (E6, E7, and E6^E7 mRNAs) to SA3358 (E4 mRNAs) or SA2709 (E2 mRNAs) (Supplementary Fig. S3A-C). However, we were unable to detect significant effects of hnRNP Q or hnRNP R on any of the alternatively spliced HPV16 early mRNAs (Supplementary Figs. S4A-J). In conclusion, hnRNP Q and hnRNP R primarily affected alternative splicing of the HPV16 late L1 mRNAs.

Taken together, our results confirm that SRSF3 and SRSF9 control HPV16 late gene expression [1, 23]. It has also been shown that the HPV infection itself alters expression of hnRNPs and SR proteins [6, 15], and SRSF3 is no exception [11]. Previously published results by Klymenko et al. demonstrated that SRSF3 is required for efficient production of the HPV16 L1 protein [11]. Another study reported that knockdown of SRSF3 increased the level of L1 mRNA with the internal exon SA3358-SD3363 [7], which is in contrast to the siRNA-mediated SRSF3 knockdown results presented by Klymenko et al. [11] and also in contrast to the results presented here. In comparison, SRSF9 showed a very clear-cut, dose-dependent induction of HPV16 late gene expression, which confirmed our previously published results [23]. SRSF3 and SRSF9 displayed the most significant induction of HPV16 late gene expression of the SR proteins investigated here, but they differed in their effect on HPV16 late L1 mRNA splicing. Finally, it remains to be determined how SRSF4 and SRSF6 reduce the levels of the alternatively spliced HPV16 L1 mRNAs as well as the significance of the modulatory effect SRSF2 and SRSF5 exert on HPV16 L1 mRNA splicing. SRSF2 and SRSF5 altered HPV16 L1 mRNA splicing but did not induce HPV16 late gene expression, suggesting that they had a more subtle effect or a modulatory role. RNA-binding proteins may also affect the stability of their target mRNAs, and it is therefore possible that the increase in HPV16 late gene expression observed here as an increase in sLuc may have been caused by an increase in HPV16 mRNA stability in addition to the effect on HPV16 mRNA splicing. Further experiments are required to establish the significance of SRSF2 and SRSF5 in the HPV16 gene expression program.

Regarding hnRNP proteins, we have previously shown that hnRNP A2 can induce HPV16 late gene expression [18], while hnRNP A1 interacts specifically with splicing silencers in the L1 coding region [26]. These results are consistent with the results presented here. Interestingly, hnRNP A1 and A2 control splicing of HPV16 and HPV18 E6/E7 mRNAs [1, 19, 27]. In the case of HPV16 E6/E7 mRNAs, it was shown that hnRNP A1 promoted production of unspliced E6 mRNA, while hnRNP A2 redirected splicing to an alternative 3’ splice site named SA742 [27]. The effect of hnRNP A1 and A2 on alternative splicing of HPV16 L1 mRNAs also differed. While hnRNP A1 promoted exclusion of the central exon marked by SA3358 and SD3632, hnRNP A2 enhanced inclusion of the same exon. In the case of hnRNP A1, there was a dose-dependent switch in the preferred splicing product, whereas in hnRNP A2, the effect was only quantitative. Despite their close relationship, hnRNP A1 and A2 were found to have distinct functions. Similarly, the effect of hnRNP G on alternative splicing of HPV16 L1 mRNAs confirmed our previously published results that hnRNP G inhibits inclusion of the central exon between SA3358 and SD3632 in L1 mRNAs [25]. hnRNP C enhanced the levels of the central-exon-containing L1 transcript in relation to the other transcripts, which is in line with previously published results [4]. Little is known about the effect of the closely related hnRNPs F and H on HPV16 mRNA splicing. Previous results have shown that hnRNP H binds to multiple binding sites in the L2 coding region, immediately adjacent to HPV16 pAE, suggesting a role for hnRNP H in HPV16 early polyadenylation [16, 17]. The results presented here demonstrate that hnRNP F and hnRNP H affected alternative splicing of the HPV16 L1 mRNAs, thereby enhancing production of HPV16 L1 mRNA. The hnRNP D proteins have previously been shown to interact with splicing silencers immediately upstream of the HPV16 splice site SD3632 and to act by inhibiting HPV16 L1 mRNA splicing [13]. Combined, the increase in unspliced L2 mRNA levels observed with hnRNP D overexpression and the concomitant reduction in spliced L1 mRNA levels are compatible with the previously observed inhibitory effect of hnRNP D on L1 mRNA splicing [13]. This is in line with the inhibitory effect of hnRNP D on HPV16 late L1 mRNA production observed here. In conclusion, our results demonstrated that the majority of the SR proteins and hnRNPs investigated here affect HPV16 late L1 mRNA levels or alternative splicing, suggesting that the majority of all hnRNPs and SR proteins have the potential to control HPV16 late gene expression.

## Supplementary Information

Below is the link to the electronic supplementary material.

Supplementary file1 (PDF 4098 KB)

## Data Availability

All data and materials described in this article are available from the corresponding author on request.
