# Supplementary material for: Identification of heterogenous nuclear ribonucleoproteins (hnRNPs) and serine- and arginine-rich (SR) proteins that induce human papillomavirus type 16 late gene expression and alter L1 mRNA splicing
Source: Arch Virol. 2021 Dec 3;167(2):563–70. doi: 10.1007/s00705-021-05317-2 (PMC8843915; doi:10.1007/s00705-021-05317-2)

**SUPPLEMENTARY INFORMATION**

**MATERIALS AND METHODS**

**Plasmids.**

The HPV16 plasmid pC97ELsL has been described previously [8, 9]. Construction of **phnRNP F phnRNP I, phnRNP A2** and **phnRNP Q** have been described previously [13] and so has **phnRNP A1** [15] and **phnRNP C1** [4]. Flag-tagged **hnRNP D**-plasmids were generously provided by Dr. R.J. Schneider [12], hnRNP G plasmid by Dr. I. C. Eperon [11], histidine and myc-tagged hnRNP L plasmid by Dr. S. Guang [5] and histidine and myc-tagged hnRNP R plasmid by Dr. P. Xu [6]. **phnRNP AB** encoding myc-tagged hnRNP AB transcript variant 1 (RC204360), **phnRNP DL** encoding hnRNP DL transcript variant 2 (RC204064) and **pRALYL** encoding myc-tagged RALYL (RC206313) were purchased from OriGene Technologies Inc. **phnRNP E1** contains the hnRNP E1 (PCBP-1) open reding frame with a FLAG-tag in the C-terminus driven by a CMV promoter. It was constructed by PCR-amplification of the hnRNP E1 coding sequence from pGEX-2TK-PCBP-1 (generously provided by Dr. H. Leffers) [7] with primers ES (5'-GGCGCGCAAGAAatggatgccggtgtgactgaaag-3') and EA (5'-CCTCGAGctaCTTATCGTCGTCATCCTTGTAATCgctgcaccccatgccc-3') followed by digestion with PteI and XhoI and subcloning into pCL086 [3]. **phnRNP K** contains the hnRNP K open reding frame with a FLAG-tag in the C-terminus driven by a CMV promoter. It was constructed by PCR-amplification of the hnRNP K coding sequence from pGEX-KT-hnRNP K (generously provided by Dr. K. Bomsztyk) [1] with primers KS (5'-GGCGCGCAAGAAatggatgccggtgtgactgaaag-3') and KA (5'-CCTCGAGctaCTTATCGTCGTCATCCTTGTAATCgctgcaccccatgccc-3') followed by digestion with PteI and XhoI and subcloning into pCL086 [3]. **phnRNP F** and **phnRNP H** contain the hnRNP F or hnRNP H open reding frames driven by a CMV promoter. They were constructed by PCR-amplification of either the hnRNP F or hnRNP H coding sequence from pET-15B-hnRNP F or pET-15B-hnRNP H (generously provided by Dr. D. Black) [2] followed by subcloning into pCL086 [3]. Plasmids expressing SR proteins have been described previously [14].

**Cells**

HeLa cells were cultured in Dulbecco's modified Eagle medium (GE Healthcare Life Science Hyclone Laboratories) with 10% bovine calf serum (GE Healthcare Life Science Hyclone Laboratories) and 1% penicillin-streptomycin (Gibco Thermo Fisher Science).

**Transfections**

Transfections of HeLa cells were carried out using Turbofect according to the manufacturer's instructions (Thermo Fisher Science). Turbofect was mixed with plasmid DNA and incubated

at room temperature for 15 min prior to drop-wise addition to 60-mm plates with subconfluent HeLa cells. Cells were harvested at 20hrs post transfection. Each plasmid was transfected in a minimum of three independent experiments.

**RNA extraction and RT-PCR**

Total RNA was extracted using TRI Reagent and Direct-zol RNA MiniPrep kit (ZYMO Research) according to the manufacturer's protocol. 1µg of total RNA were reverse transcribed in a 20 µl reaction at 37°C by using M-MLV Reverse Transcriptase (Invitrogen) and random primers (Invitrogen) according to the protocol of the manufacturer. One microliter of cDNA was subjected to PCR amplification. HPV16 late L1 mRNAs were amplified with RT-PCR primers 97S or 773S and L1A (**Supplementary Table T1**). HPV16 mRNAs spliced from HPV16 5'-splice site SD226 to 3'-splice sites SA409, SA526, and SA742 were PCR amplified with RT-PCR primers 97S and 880A (**Supplementary Table T1**). HPV16 mRNAs spliced from 5'-splice site SD880 to 3'-splice sites SA2709 or SA3358 were amplified with RT-PCR primers 773S and E2A or E4A (**Supplementary Table T1**). GAPDH cDNA was amplified with primers gapdhf and gapdhr (**Supplementary Table T1**).

**Secreted luciferase assay**

The *Metridia longa* secreted luciferase activity [10] in the culture medium of the C33A2 cells was monitored with the help of the "Ready To Glow" secreted luciferase reporter assay according to the instructions of the manufacturer (Clontech Laboratories). Briefly, 50 ul of cell culture media were mixed with 5 ul of secreted luciferase substrate in reaction buffer, and the luminescence was monitored in a Tristar LB941 luminometer (Berthold Technologies).

**Quantitations.**

The softeware used to determine band intensity in RT-PCR gels is "Image Lab 6.0.1" and quantitations were performed with the software "Prism GraphPad 8.4.0".

**REFERENCES**

1. Bomsztyk K, Van Seuningen I, Suzuki H, Denisenko O, Ostrowski J (1997) Diverse molecular interactions of the hnRNP K protein. FEBS Lett 403:113-115
2. Chou MY, Rooke N, Turck CW, Black DL (1999) hnRNP H is a component of a splicing enhancer complex that activates a c-src alternative exon in neuronal cells. Mol Cell Biol 19:69-77
3. Collier B, Öberg D, Zhao X, Schwartz S (2002) Specific inactivation of inhibitory sequences in the 5' end of the human papillomavirus type 16 L1 open reading frame

results in production of high levels of L1 protein in human epithelial cells. J Virol 76:2739-2752

4. Dhanjal S, Kajitani N, Glahder J, Mossberg AK, Johansson C, Schwartz S (2015) Heterogeneous Nuclear Ribonucleoprotein C Proteins Interact with the Human Papillomavirus Type 16 (HPV16) Early 3'-Untranslated Region and Alleviate Suppression of HPV16 Late L1 mRNA Splicing. J Biol Chem 290:13354-13371
5. Guang S, Felthauser AM, Mertz JE (2005) Binding of hnRNP L to the pre-mRNA processing enhancer of the herpes simplex virus thymidine kinase gene enhances both polyadenylation and nucleocytoplasmic export of intronless mRNAs. Mol Cell Biol 25:6303-6313
6. Huang J, Li SJ, Chen XH, Han Y, Xu P (2008) hnRNP-R regulates the PMA-induced c-fos expression in retinal cells. Cell Mol Biol Lett 13:303-311
7. Leffers H, Dejgaard K, Celis JE (1995) Characterisation of two major cellular poly(rC)-binding human proteins, each containing three K-homologous (KH) domains. Eur J Biochem 230:447-453
8. Li X, Johansson C, Cardoso-Palacios C, Mossberg A, Dhanjal S, Bergvall M, Schwartz S (2013) Eight nucleotide substitutions inhibit splicing to HPV-16 3’-splice site SA3358 and reduce the efficiency by which HPV-16 increases the life span of primary human keratinocytes. PLoS One 8:e72776
9. Li X, Johansson C, Glahder J, Mossberg AK, Schwartz S (2013) Suppression of HPV-16 late L1 5'-splice site SD3632 by binding of hnRNP D proteins and hnRNP A2/B1 to upstream AUAGUA RNA motifs. Nucleic Acids Res 22:10488-10508
10. Markova SV, Golz S, Frank LA, Kalthof B, Vysotski ES (2004) Cloning and expression of cDNA for a luciferase from the marine copepod *Metridia longa*. J Biol Chem 279:3212-3217
11. Nasim MT, Chernova TK, Chowdhury HM, Yue BG, Eperon IC (2003) HnRNP G and Tra2beta: opposite effects on splicing matched by antagonism in RNA binding. Hum Mol Genet 12:1337-1348
12. Sarkar B, Lu JY, Schneider RJ (2003) Nuclear import and export functions in the different isoforms of the AUF1/heterogeneous nuclear ribonucleoprotein protein family. J Biol Chem 278:20700-20707
13. Somberg M, Zhao X, Fröhlich M, Evander M, Schwartz S (2008) PTB induces HPV-16 late gene expression by interfering with splicing inhibitory elements at the major late 5’-splice site SD3632. J Virol 82:3665-3678
14. Tranell A, Fenyö EM, Schwartz S (2010) Serine- and arginine-rich proteins 55 and 75 (SRp55 and SRp75) induce production of HIV-1 vpr mRNA by inhibiting the 5'-splice site of exon 3. J Biol Chem 285:31537-31547

15. Zheng Y, Jonsson J, Hao C, Shoja Chaghervand S, Cui X, Kajitani N, Gong L, Wu C, Schwartz S (2020) Heterogeneous Nuclear Ribonucleoprotein A1 (hnRNP A1) and hnRNP A2 Inhibit Splicing to Human Papillomavirus 16 Splice Site SA409 through a UAG-Containing Sequence in the E7 Coding Region. J Virol 94:e01509-01520

Supplementary Table T1. RT-PCR primers.

| Primer name | Sequence (5′ - 3′) |
|---|---|
| 97S | GTCGACCTGCAATGTTTCAGGACCC |
| 773S | GCACACACGTAGACATTCGTACTTTG |
| 880A | GATCAGCCATGGTAGATTATGGTTTC |
| E2A | CCTGACCACCCGCATGAACTTCC |
| E4A | TGCTGCCTAATAGTTTCAGGAGAGG |
| L1A | GCAACATATTCATCCGTGCTTACAACC |
| FSet3 | GCACCCCCTTTAACAGTAGATCC |
| RSet3 | TACAGATGGGTCAGTGAAAGTG |
| gapdhf | ACCCAGAAGACTGTGGATGG |
| gapdhr | TTCTAGACGGCAGGTCAGGT |

**SUPPLEMENTARY FIGURE LEGEND.**

**SUPPLEMENTARY FIGURE S1. (A)** Schematic representation of the HPV16 genome and **(B)** the HPV16 subgenomic pC97ELsL reporter plasmid. Transcription of the HPV16 sequences in the pC97ELsL plasmid is driven by the human cytomegalovirus immediate early promoter (CMV). HPV16 splice sites are indicated. Numbers refer to the HPV16 reference strain HPV16R (GeneBank: K02718.1). Early and late polyadenylation signals pAE and pAL are indicated. p97 and p670 indicate HPV16 early and late promoters, respectively. IRES, poliovirus 2A internal ribosome entry site; sluc, secreted luciferase. (**C**) Alternatively spliced HPV16 L1 mRNAs produced by subgenomic HPV16 expression plasmid pC97ELsL are indicated. Arrows indicate positions of RT-PCR primers. (**D**, **E**) RT-PCR on RNA extracted from HeLa cells transfected with pC97ELsL in the absence (-) or presence of the indicated hnRNP D expression plasmids. RT-PCR was performed with primers FSet3 and RSet3 in the presence (**D**) or absence of RT **(E)**.

**SUPPLEMENTARY FIGURE S2.** Secreted luciferase (sLuc) activity in the cell culture medium of HeLa cells transfected with pC97ELsL in the absence (-) or presence of 2-fold serially diluted phnRNP Q (2.0ug, 1.0ug, 0.5ug and 0.25ug) **(A)** or 2-fold serially diluted phnRNP R (2.0ug, 1.0ug, 0.5ug and 0.25ug) **(B)**. RT-PCR on RNA extracted from HeLa cells transfected with pC97ELsL in the absence (-) or presence of 2-fold serially diluted phnRNP Q (2.0ug, 1.0ug, 0.5ug and 0.25ug) **(C)** or 2-fold serially diluted phnRNP R (2.0ug, 1.0ug, 0.5ug and 0.25ug) **(D)**. RT-PCR was performed with RT-PCR primers 773S and L1A. The HPV16 splice sites used to generate the mRNAs amplified by the RT-PCR primers are indicated to the right. Gapdh cDNA was PCR-amplified by primers gapdhf and gapdhr. M, molecular size marker. **(E)** Quantitation of RT-PCR bands in (C). **(F)** Quantitation of RT-PCR bands in (D).

**SUPPLEMENTARY FIGURE S3. (A)** Schematic representation of the HPV16 subgenomic pC97ELsL reporter plasmid. Transcription of the HPV16 sequences in the pC97ELsL plasmid is driven by the human cytomegalovirus immediate early promoter (CMV). HPV16 splice sites are indicated. Numbers refer to the HPV16 reference strain HPV16R. Early and late polyadenylation signals pAE and pAL are indicated. IRES, poliovirus 2A internal ribosome entry site; sLuc, secreted luciferase. **(B, C)** Schematic representation of HPV16 early mRNAs produced by pC97ELsL. Numbers represent HPV16 splice sites. RT-PCR primers 97S, 773S, 880A, E2A and E4A are indicated.

**SUPPLEMENTARY FIGURE S4.** RT-PCR on RNA extracted from HeLa cells transfected with pC97ELsL in the absence (-) or presence of 2-fold serially diluted phnRNP Q (2.0ug, 1.0ug, 0.5ug and 0.25ug) **(A-E)** or 2-fold serially diluted phnRNP R (2.0ug, 1.0ug, 0.5ug and 0.25ug) (**G-J**). RT-PCR was performed with the indicated primer pairs. The HPV16 splice sites used to generate the mRNAs amplified by the RT-PCR primers are indicated to the right. Gapdh cDNA was PCR-amplified by primers gapdhf and gapdhr. M, molecular size marker.

Supplementary Figure 1

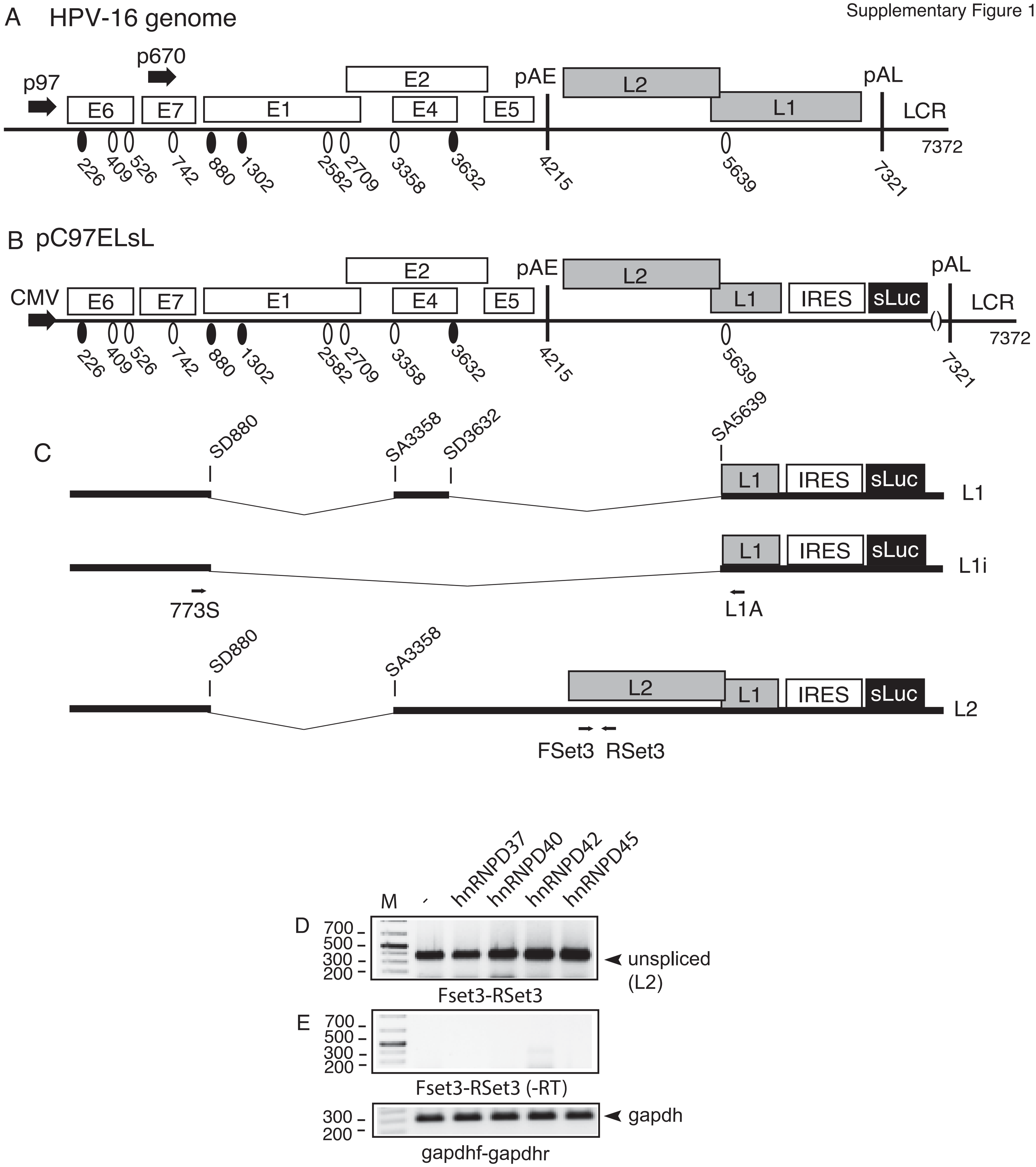

Supplementary Figure S2

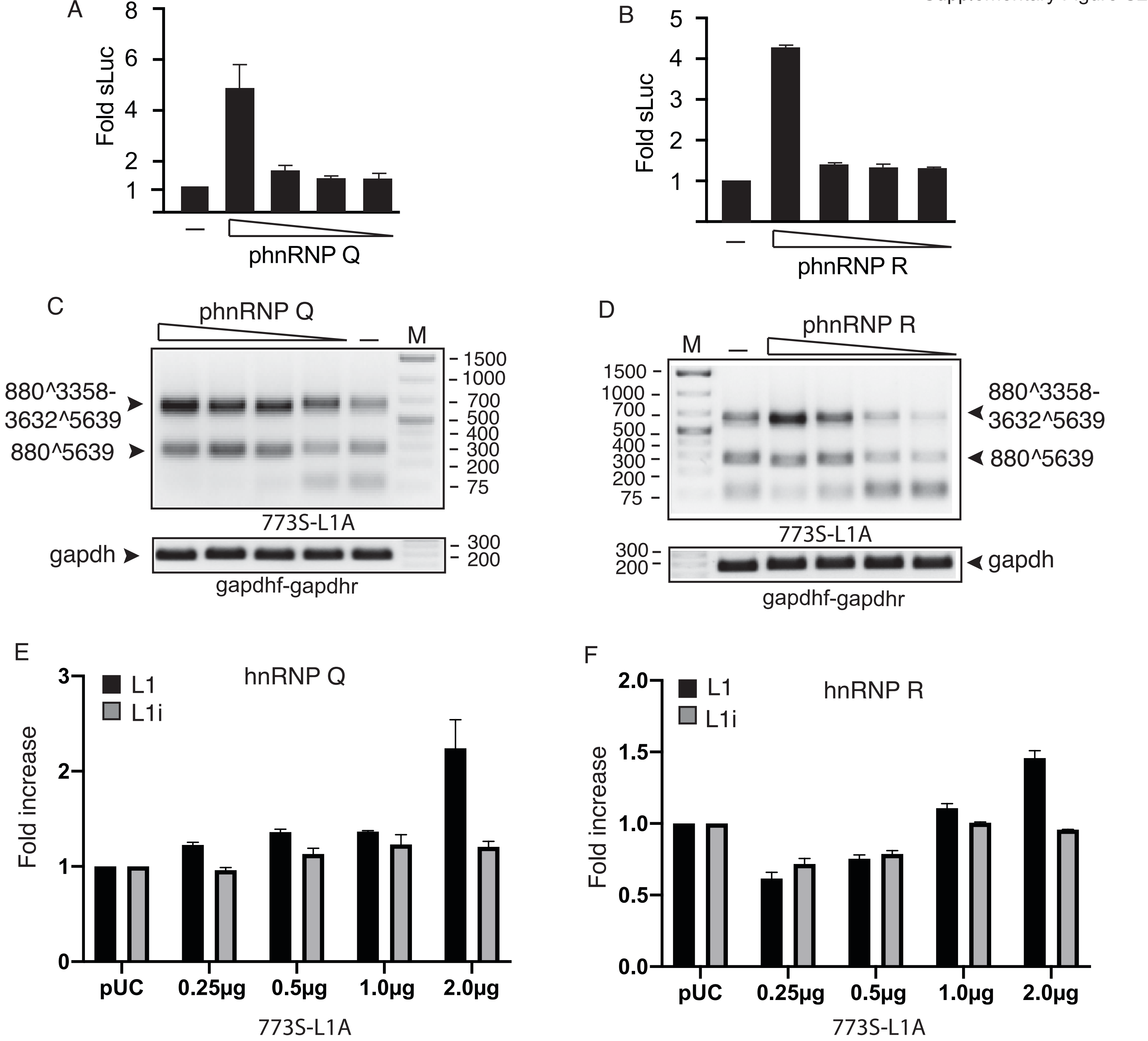

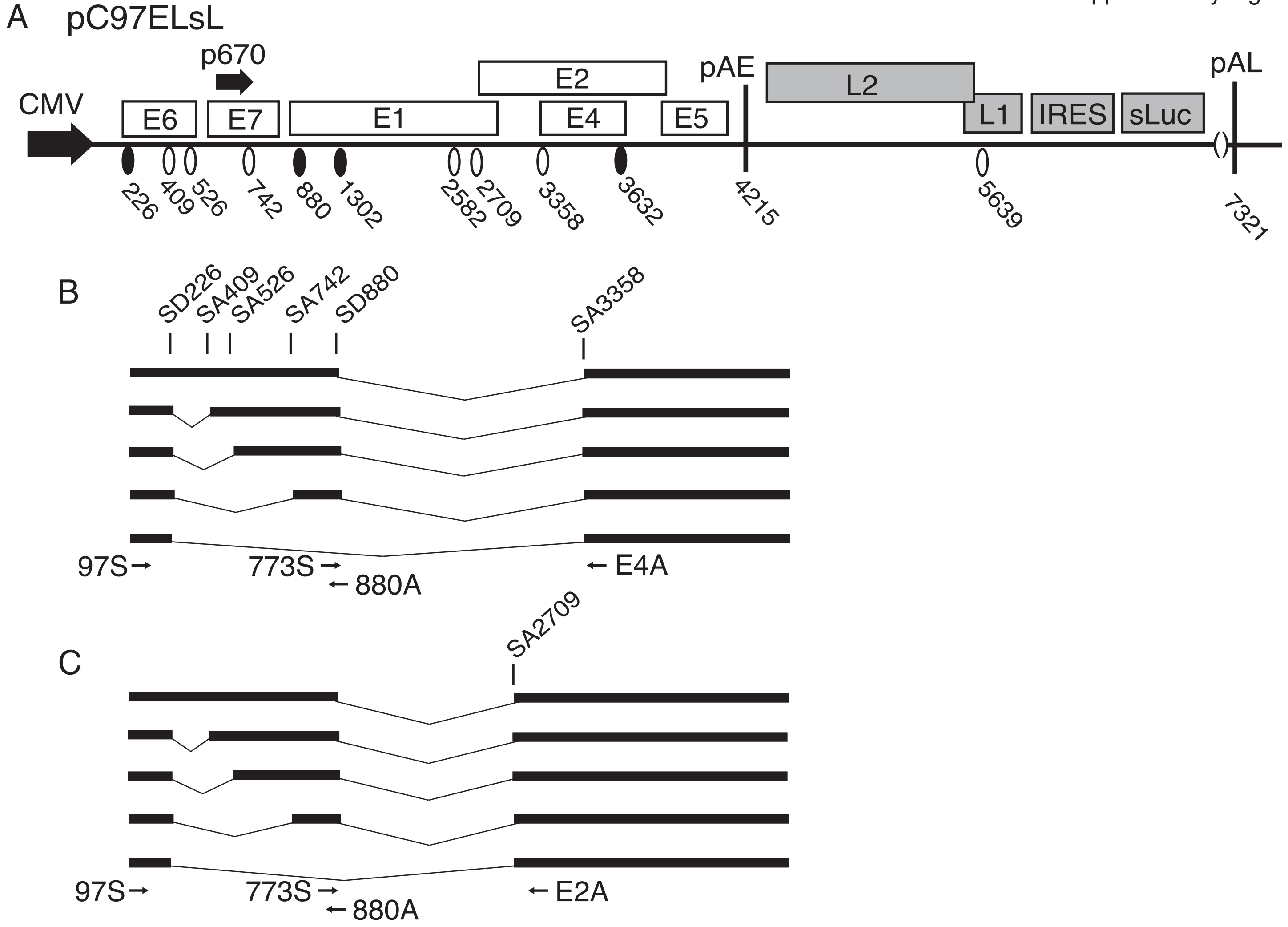
A
pC97ELsL
p670
CMV
E6
E7
E1
E2
E4
E5
pAE
L2
L1
IRES
sLuc
pAL
226
409
526
742
880
1302
2582
2709
3358
3632
4215
5639
7321
B
SD226
SA409
SA526
SA742
SD880
SA3358
97S
773S
880A
E4A
C
SA2709
97S
773S
880A
E2A

Supplementary Figure S4

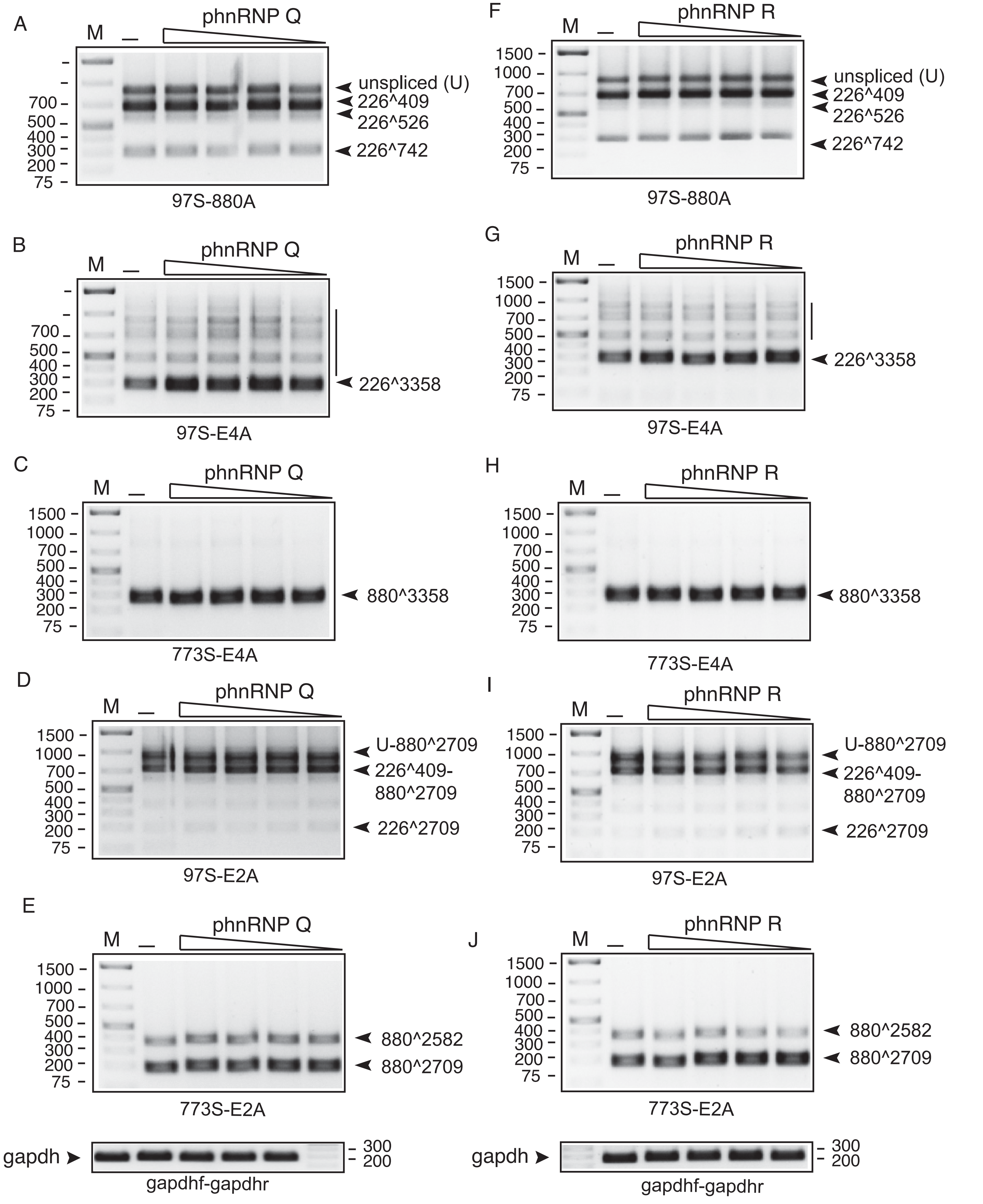

Supplement: Supplementary file 1 — Supplementary file1 (PDF 4098 KB) [file 705_2021_5317_MOESM1_ESM.pdf]
